# Improving Understanding of Participation and Attrition Phenomena in European Cohort Studies: Protocol for a Multi-Situated Qualitative Study

**DOI:** 10.2196/14997

**Published:** 2020-07-15

**Authors:** Sandra C S Marques, Julia Doetsch, Anne Brødsgaard, Marina Cuttini, Elizabeth S Draper, Eero Kajantie, Jo Lebeer, Sylvia van der Pal, Pernille Pedersen, RECAP - WP6 QS Work Group, Henrique Barros

**Affiliations:** 1 EPIUnit Instituto de Saúde Pública da Universidade do Porto Universidade do Porto Porto Portugal; 2 Centro em Rede de Investigação em Antropologia Instituto Universitário de Lisboa Lisbon Portugal; 3 School of Public Health and Primary Care Faculty of Health, Medicine and Life Sciences Maastricht University Maastricht Netherlands; 4 Department of Paediatrics and Adolescent Medicine Copenhagen University Hospital Amager Hvidovre Copenhagen Denmark; 5 Research Unit of Perinatal Epidemiology, Clinical Care and Management Innovation Research Area Ospedale Pediatrico Bambino Gesù Institute for Research Hospitalization and Health Care Rome Italy; 6 Department of Health Sciences College of Life Sciences University of Leicester Leicester United Kingdom; 7 Public Health Promotion Unit National Institute for Health and Welfare Helsinki Finland; 8 PEDEGO Research Unit MRC Oulu University of Oulu and Oulu University Hospital Oulu Finland; 9 Department of Clinical and Molecular Medicine Norwegian University for Science and Technology Trondheim Norway; 10 Children’s Hospital Helsinki University Hospital Helsinki Finland; 11 Department ELIZA Primary & Interdisciplinary Care Faculty of Medicine & Health Sciences University of Antwerp Antwerp Belgium; 12 TNO Child Health Leiden Netherlands; 13 see Acknowledgments; 14 Departamento de Ciências da Saúde Pública e Forenses e Educação Médica Faculdade de Medicina Universidade do Porto Porto Portugal

**Keywords:** European cohorts, VPT, VLBW, preterm, participation, attrition, multi-situated qualitative study, collaborative visual methods, triangulation

## Abstract

**Background:**

Cohort studies represent a strong methodology for increasing one’s understanding of human life-course development and etiological mechanisms. Retention of participants, especially during long follow-up periods, is, however, a major challenge. A better understanding of the motives for participation and attrition in cohort studies in diverse sociogeographic and cultural settings is needed, as this information is most useful in developing effective retention strategies.

**Objective:**

This study aims to improve our understanding of participation and attrition phenomena in a European cohort study of very preterm/very-low-birth-weight (VPT/VLBW) infants from various sociogeographic and cultural settings to better understand variability and ultimately contribute to developing novel and more “in-context” strategies to improve retention.

**Methods:**

This study uses a triangulation of multisituated methods to collect data on various cohorts in the Research on European Children and Adults Born Preterm (RECAP) network, which include focus group discussions, individual semidriven interviews, and a collaborative, reflexive visual methodology (participant-generated VideoStories) with relevant key actors involved with these cohort studies such as adult participants, parents (caregivers), cohort staff, health care professionals, and academic researchers. The methodological strategy aims to provide a shared flexible framework of various qualitatively driven methods to collect data on VPT/VLBW adult and child cohorts, from which research partners may choose and combine those most pertinent to apply in their own specific contexts. Data from all sources and sites will be submitted to a triangulation of phenomenological thematic analysis with discourse analysis.

**Results:**

As of January 2020, in this study, we enrolled 92 participants variously involved with child and adult RECAP partnering cohorts from six countries. Multisite enrollment and data collection are expected to be completed in all seven study settings by June 2020. Findings will be reported in future publications.

**Conclusions:**

Qualitative research methods are a useful complement for enriching and illuminating quantitative results. We expect that opting for a multisituated study approach addressing the interplay of the lived experience of individuals in both researcher and researched stances of particular cohort study settings will contribute to filling some gaps in the understanding of participation variability and effectiveness of different implemented strategies in context. Moreover, health research subjects have traditionally been positioned as passive objects of study rather than active participants, even though they have the greatest stake in improving health care policies and practices. Including collaborative methods allows us to counteract the “top-down” model by handing over some research control to the very people who are providing the data on which research findings will be based while also acknowledging the value of their involvement.

**International Registered Report Identifier (IRRID):**

DERR1-10.2196/14997

## Introduction

### Background and Rationale

The Horizon 2020 Project Research on European Children and Adults Born Preterm (RECAP), funded by the European Union (under grant agreement no. 733280; January 2017- March 2021), brings together 20 population-based cohorts from 13 European countries, with individuals born very preterm (VPT: <32 weeks of gestation) or with very low birth weight (VLBW: <1500 g), who were followed up since birth. The network includes cohorts assembled over a period of 4 decades, presently covering a large age span, from childhood (>7 years old) to early adulthood (<40 years old). The overall aim of RECAP is to improve health, development, and quality of life of children and adults born VPT/VLBW, by developing collaborative multidisciplinary research on these geographically and temporal diverse cohorts and optimize their use for innovation in practice and policy.

Cohort studies are a strong methodological approach to understand human life-course development and causal mechanisms. A major issue in long-term follow-up studies, however, is that response decreases with a lapse of time between recruitment and follow-up assessments. It is not clear how this attrition influences the results of follow-up studies, different research cases point to distinct outcomes [1], it is certain that they become prone to considerable selection biases with losses to follow-up and dropouts as little as 20% [2]. Cohorts are complex research structures that require sustained involvement of participants, professionals, funding, and supporting infrastructure to ensure continued attention to timeliness, attrition, and quality of collected information. Those requirements are indispensable to meet high scientific standards and allow appropriate translation of findings into clinical practice and policy action. Retention of participants is a major concern and a well-known challenge, and due to international specificities in research regulation and contextual differences, approaches to these issues will also necessarily vary.

Available evidence suggests that investigators should consider using multiple strategies to maximize the retention of participants and that the use of incentives is associated with an increase in retention, which improves with greater incentive value [3-5]. In 2011, the widely quoted systematic review of Booker, Harding, and Benzeval [6] concluded that the use of financial incentives was associated with an increase in retention rates (we note that the expression “rate” is kept here as used in referred sources). Whether cash was the most effective incentive was not clear from studies that compared cash and gifts of similar value. Relevant increases in retention were also found for posting repeat questionnaires, using reminders as well as offering alternative locations and modes of data collection [6]. Other studies also point out that the use of targeted strategies, such as incentives to nonresponders from previous waves of the study, is a cost-effective approach to retain participants at high risk of dropping out and that regular contact between participants and investigators enhances bonding and helps ensure enduring identification with the study [7,8].

These are useful insights about how retention can be enhanced by combining a number of strategies and bonding tools. It should be noted, however, that most available sources on more generalizable results, such as systematic reviews, are yet constrained by the small number, geographical concentration, scarce details, and inconsistent description of published studies reporting implemented strategies. Inferential leaps or generalization to other contexts and, subsequently, usefulness of retention strategies investigated may differ. Further primary research is needed to expand the population assessed and diversity of study settings, especially in Europe. Moreover, there is a paucity of literature regarding expectations and motives for participation and for participants’ reluctance to continue in cohort studies, although such information is essential to enhance recruitment and improve retention [9-11].

To improve understanding of the context of behaviors, survey research, broadly referring to questionnaires and structured interviews to obtain quantifiable aggregated data, provides estimates of many variables. However, survey results are only as good as the questions asked [12] and the potential of the data generated is limited to the assumptions framing the response options. A qualitative research approach is therefore a useful complement for enriching and enlivening quantitative results. Some of the distinctive features of this kind of approach are the use of open, exploratory research questions and meaning-based rather than statistical forms of data analysis, which tend to potentiate the emergence of something new [13-16]. We expect that opting for a triangulation of qualitative methods to garner feedback from various parties involved with diverse cohorts’ settings will contribute toward filling some gaps in the understanding of participation and attrition phenomena.

### Overall Aim and Objectives

The overall aim of this study is to provide an insight into participation and attrition phenomena in VPT/VLBW cohort studies in various sociogeographic, linguistic, and cultural settings in order to increase our understanding of variability and to ultimately contribute to developing novel and more “in-context” strategies to improve retention. It is a direct response to RECAP’s concerns toward improving data collection, follow-up, and participant involvement in cohort studies.

Following the epistemological principle of valuing “in-context” and nuanced inside knowledge from various parties, multisite data collected for this study will contribute to the following objectives:

- To explore perceptions, feelings, and expectations of parents (caregivers) of children and adults born VPT/VLBW who participate in European cohorts studies about enrollment and continuity of participation.
- To explore the experiences and practices of diverse key actors involved with cohort studies with regard to the activities, procedures, and difficulties faced that may affect participants’ attendance and commitment continuity to the study.
- To investigate the interplay between lived experience and expectations of cohort participants and the manifested responses obtained by the professionals in follow-up enquiries in different European studies.

## Methods

### Setting and Sampling

The study proposes a triangulation of multisituated methods to collect data on RECAP’s cohorts in various European countries, which include focus group interview, individual semidriven interview, and a collaborative reflexive visual methodology. Our methodological strategy provides a shared framework of various qualitatively driven methods to collect data, from which research partners may choose and combine those most appropriate to their own particular contexts. A multisituated approach not only comprises the concept of multisites or multilocations [17], but also assumes the significance of situated knowledge. It stresses the material, social, and political conditions that contribute to gaining (multiple, partial, diverse) knowledge, and the responsibility to consider them just as valuable [18,19]. Given the framework constraints and nature of the phenomena under study (eg, variety of cohort studies and settings), we find it the most promising strategy to produce rich multimodal and original data, which include diverse subjects’ voices, perceptions, and experiences.

As the study is focused on garnering feedback from various parties involved in cohorts of RECAP’s network, potential participants of this study will be found, contacted, and enrolled with the collaboration of partnering cohort teams. The range of participating cohorts will achieve a wide sociogeographic heterogeneous sample inclusive of parents of children cohorts and of cohort participants aged 18 years and over; health care professionals; and other relevant key actors such as current and former staff cohort members, representatives of parent organizations, and academic researchers involved with VPT/VLBW cohort studies in various European countries. The sample should be varied enough to obtain feedback for most experiences and perceptions in both research stances. In order to satisfy the saturation criterion, a purposive (selective) nonprobability sampling strategy will be used. The intended total sample size is 120-130 participants to be recruited through a range of multisituated methods from 7 to 8 different cohort settings and countries. Accordingly, local protocols, setting, methods, and sample determination in all sites will be discussed and finalized with the study coordinator.

### Data Collection

To be eligble, RECAP’s partnering cohorts have to identify a researcher/translator, who can translate content into English, with experience in qualitative research and methods to carry out data collection locally and meet the requirements to conduct one or more of the following proposed methodologies within the cohort of its scope:

1. One or two focus groups with professionals or other relevant key actors involved with the cohort, for a 1- to 1.5-hour discussion. These discussions are conducted either in the native speakers’ language or English (in case all potential participants are proficient English speakers and the local researcher finds it appropriate).
2. One or two focus groups with parents (caregivers) of children or focus groups with adults who participate in the cohort, for a 1- to 1.5-hour discussion. These discussions are conducted in the native speakers’ language. In case of groups comprising foreign are, immigrants, or languages’ participants speaking different languages, the local researcher will choose the most inclusive language to conduct the meeting.
  When the approach to nonresponders from previous waves of the study is available, particular effort will be placed in inviting them to participate, stressing that this initiative has the specific purpose of hearing from them about their difficulties, constraints, and suggestions in order to find more adequate strategies to meet their expectations. Partners may implement further incentive strategies to foster participation, in case they find it appropriate.
  Focus groups will attempt to include 5-7 participants, a number large enough to generate a variety of perspectives and small enough to allow every participant to engage in the exploratory discussion in greater depth [20-22]. Focus group discussions will be driven by 6-8 key issues commonly defined to approach the phenomena under study while including some in-context subtopics of discussion elected by local partners as relevant to the specificities of the cohort study and their particular participants (see Multimedia Appendix 1). The exploratory approach chosen by this study aims to potentiate the emergence of something new from the discussions, and therefore, the moderator will be as nondirective as possible and make use of the guide of key issues to approach only as discussion triggers and if not spontaneously approached by participants. These discussions will also be used to explore the “territory” and map key issues for further group or individual semidriven interviews.
  In cases where the cohort participants live far apart or are more responsive to novel digital tools, online focus groups on a secured forum can be considered. Participants in the forum can either express an unlimited number of comments on each of the defined 6-8 key issues to approach over the course of a week or react to one key issue under discussion per day. The advantage is that participants can express themselves at their own convenience while incorporating and reacting to the opinions of others. This often results in less spontaneous interaction among participants, but more focused output. For the particularities of this mediator, in order to balance the variety of perspectives and engagements generated in the discussion, the number of participants suggested is 15-20 [23].
  In cases where focus group discussions were considered not feasible, or failed to reach saturation for one or more key issues, or where a willing participant is only available to be interviewed separately, individual semidriven interviews may be conducted face-to-face, through videoconference or teleconference.
3. Individual semidriven interviews are conducted in the native speakers’ language for no more than 1 hour. These interviews will be driven by the same 6-8 key issues commonly defined to approach the phenomena under study while including some in-context subtopics of discussion selected by local partners as relevant to the specificities of the cohort study and their particular participants.
4. Participant-generated VideoStories, a collaborative visual methodology, with 4-7 cohort participants aged 18 years and over or with 4-7 parents (caregivers) of children participating in the cohort.
  Photo and video elicitation are qualitative methods of investigation in which images are used to facilitate meaningful responses. Still images or video can be taken by the researcher, pulled from a third-party source, or generated by the research participants. In participant-generated visual methodologies, the participant is the one who generates the (audio)visual images that will be further analyzed. These are used to elicit ethnographic data and less-driven personal views while maximizing the alternatives for individual expression and representation.
  The collaborative methodology chosen for this study is derived from photo/video voice process [24,25]. It is a methodology well suited for a phenomenological analysis focused on the meaning of behavior, narrative, and “lived personal experience” [26]. Using collaborative techniques that combine video-generated images and critical dialogue, participants are expected to reflect in-depth on the phenomena under study and communicate their concerns to represent their personal experience and acquired knowledge, as research participants expose individual and social problems and ignite changes in behavior. Both the researcher and the participants benefit. On one hand, participants are encouraged to use their critical, confessional, and authoritative/pedagogical voices to clearly establish themselves as legitimate partners in the study [27,28]. On other hand, through reflexivity, they come to new understandings of their practices and behaviors, prompting some to change or develop new strategies [29-31], potentially increasing their identification with the cohort studies and retention. It is also particularly advantageous to prompt recall from multiple subjects concerning their life events that the researcher cannot join as a participant observer.
  The VideoStories methodological process involves two steps:
  - “VideoStories assignment”: In order to collect audiovisual data that can be compared and contrasted across participants, the participants will be given a standardized assignment to record during a similar visual narrative period. Each participant will agree to generate a set goal of 3-4 videos, each with a maximum length of 10 minutes (4-6 minutes ideally), shot on different days, within a 1-week period. This will encourage the participant to reflect in advance on what, how, and when to shoot each video.
  - “VideoStories debriefing conversation”: During week 2, participants handover their photographs/videos to the researcher. Copies of video files will be transferred to the researcher and secured in a protected dedicated storage. Each participant will meet with the researcher at a mutually convenient place for the VideoStories debriefing conversation. This is a semidriven interview or image-elicitation interview [32-34], about the videos, one by one, which should take no more than 1-1.5 hours.
  In the European context, the mobile phone has replaced other digital recording devices in the daily lives of a large proportion of the population, crossing economic classes and age groups, which converts this technology into one of the most suitable tools for self-expression and documentation. In addition to being familiar daily tools, mobile phones are also easier to manipulate than video cameras for basic video production. Since participant-generated images in this study are only intended for elicitation and will not be published or disseminated in any form, no further training in video editing will be needed. For these reasons, and anticipating that most of the willing participants will have their own mobile phones capable of producing video images (minimizing the investment in equipment acquisitions and training), we propose to use this technology to generate VideoStories. In case a willing participant does not have his/her own device, he/she will be given access to video equipment.

### Data Analysis

Data collected from multiple sites through a variety of proposed methods will enable triangulation of data, comparing and contrasting data from a range of sources and perspectives, thus enhancing a more nuanced understanding of the phenomena under study.

Interview data from all sources will be audio recorded, transcribed, and translated into English by multisite partnering researchers. It will then be used for triangulation of phenomenological thematic analysis with discourse analysis. Phenomenological analysis is an approach to qualitative research with an idiographic (representational) focus, which means that it aims to offer insights into how a given person, in a given context, makes sense of a given phenomenon. As mentioned earlier, it is focused on the whys; the meaning of behavior, narrative, and “lived personal experience,” and has its theoretical origins in phenomenology and hermeneutics. Following Edmund Husserl and Wilhelm Dilthey’s hermeneutics, the concept of “lived personal experience,” in which experience constitutes the primary reality, includes not only behavior, acts, and sentiments but also reflection of these on the inner, personal experience [26,35].

Both visual and verbal depictions will be treated as narratives and the focus is on a deep understanding of the meaning of description. The meanings, usually implicit, need to be made explicit with thematic analysis. The research team performing the analysis will look then for recurrent themes and repetitions (discursive formations) to determine if any patterns (or representational axes) emerged. Usually, there are two types of themes, collective themes that occur across contexts and groups of participants having a similar experience, and individual themes that are unique to a particular context or a few individuals. Final interpretative analysis will emerge by the generic application of the mode of contents contingency. Contingency derives from Foucault’s definition of discursive formation. It is the process of finding the regularity in discourse dispersion.

Data will be sorted (“coded”) and then categorized by hand by the research team performing the analysis and verified by the local researchers involved with collection and translation of multisite data. As the study will generate a large body of data, NVivo2011 (a computer-assisted qualitative data analysis software) may be used to handle and help highlighting similarities and differences across the subsets of data for some specific circumstances. However, the first principle of analysis of phenomenological data is to use an emergent strategy, to allow the method to follow the nature of the data itself, which may emerge or change in the course of analysis. In phenomenological thematic analysis and discourse analysis, we abstract themes, discursive formations, and representational axes, and we do not use “a priori coding” instrumentation. For that reason, computer analysis support may only take a marginal role.

### Ethics and Dissemination

Despite some variation in the ways ethical approval practices work across diverse social sciences research sectors, the issues assessed by the ethics committees are common concerns to all researchers and include items such as avoidance of harm, researcher integrity, honesty, voluntary informed consent, confidentiality of information provided by participants, and anonymity. However, the general nature of these professional codes and guidelines means that the ethical issues relating to visual methods are not specifically addressed within most codes. Concerns have been expressed among researchers that study designs with a visual element may sometimes be seen with distrust by ethical reviewers based only on unfamiliarity with the methodology [36,37]. Yet, most ethical issues raised by visual research are, arguably, the ones that are relevant to all research. It is mainly the specific issue of dissemination of identifiable images of individuals (and places) that presents the most significant specific ethical challenge to manage in visual research.

For visual research methods, it is important to consider consent as pertaining to not just to the collection of images but also processing and analysis, presentation, and dissemination of images. In the light of this, it is advisable to request that interviewees give consent to the fair handling of records to the researcher. In the case of study participant—generated audio-visual data, copyright rests with the respondents. The researcher may only use the data as agreed with the author, while the author may use and reuse them at his/her wish. While legally the video or photograph taker owns the image and can assign copyright or partial rights of use to the researcher if they wish to do so, other people depicted in the images have not necessarily given their consent to the image. Participants are therefore encouraged to avoid the depiction of other persons in their photographs/videos. In case they include other persons (eg, their children or other family members), they are reminded to always ask their permission, not shoot images that could be embarrassing or troubling, and obtain their assent/written consent for appearance release. Without fulfilling that requirement, those images would not be processed by the researcher.

For this study, neither researcher-generated nor participant-generated audio and visual images will be released for publication or dissemination. Therefore, consent will be sought to participate in the research study and for the use and processing of any generated audio or image records for elicitation of information only within the research team for analysis purposes. Publications and presentations from the study will display findings anonymously (names, recorded utterances, and other personally identifiable information will not be used). Study participants will be given information about the project orally as well as in written form. The methodological design used in this study ensures that all prospective participants will have the opportunity to discuss with the researchers confidentiality and data security procedures as well as who will see their information, before signing the informed consent. We also believe that creating processes of ongoing consent offers a useful way of respecting the participants’ wishes. It safeguards the notion that if participants want it, they can withdraw or propose changes to the limits of their given consent at any time. If a participant decides to withdraw from the study, from that date, the researchers will not use the information already provided by the participant for any further analysis or publications.

All researchers are also subject to legislation on data protection, which demands that data are stored securely and do not lead to any breach of confidentiality and anonymity. All generated data within this study will be secured in a protected dedicated storage and kept for a period of 5 years after the completion of the RECAP Project. As data will be collected from different European countries and handled by members of the research team located in those countries, a secured access policy based on a need-to-know basis principles and password protection for electronic data will be implemented (ie, only the users who need to access the data will be allowed to do so). Any assisting nonresearcher interpreters-translators and transcribers to this study will also sign a confidentially agreement. When research records are to be destroyed, participants’ confidentiality will be kept throughout the process.

In all fieldwork sites, approval by ethics committees, data-protection authorities, and signed informed consents by responders in their spoken languages will be sought according to national rules. Additional measures to protect privacy may be applied by the regions according to national rules and requirements by local ethics committees.

## Results

This paper focuses on the methodological research approach used for this study and, therefore, the findings will be reported in future publications.

The implementation of this study protocol involves multiple resources for data collection from various cohort structures, research institutions, and countries, requiring collaboration between many involved parties. Due to international differences in the regulation of research, diversity of cohorts, and particular constraints of local research teams involved, it was defined a flexible schedule for the multi-situated implementation of the study.

As of January 2020, ethical clearance was obtained for seven sites, three cohort studies of children in Denmark, Italy, and Portugal and four cohort studies with participants aged 18 years and over in Belgium, Finland, the Netherlands, and Norway. A total of 92 participants variously involved with partnering cohorts of children and adults born VPT/VLBW from six countries were already enrolled to this study. To date, the total sample comprises 29 professionals variously involved with cohorts in five countries (including former and current cohort staff members, health care professionals, and researchers involved with these and other cohort studies), 26 parents of children participating in cohorts are from three countries (of which nine had failed to respond in one or more waves of the studies), and 37 adult participants in cohorts from two countries. Multisite enrollment and data collection are expected to be completed in all seven study settings by June 2020. Although subsets of data will be handed over for analysis at a different pace, results are expected to be published by the end of 2020.

As described earlier, following the epistemological principle of valuing situated knowledge, decision on data collection methods was enabled by partnering researchers’ understanding of their own particular contexts. By way of example, in the Netherlands, adult participants in the Project on Preterm and Small for Gestational Age Infants cohort were able to choose to be interviewed by telephone, videoconference, or taking part in online focus groups on a secured forum; whereas in Portugal, all proposed methodologies were implemented face-to-face and the researchers traveled to alternative locations to meet parents of children participating in the Effective Perinatal Intensive Care in Europe/Screening to Improve Health in Very Preterm Infants in Europe cohort studies.

## Discussion

In spite of the knowledge gained on participation and attrition phenomena from systematic reviews, in-depth examination of retention strategies, of how these were modified and adapted over the cohorts’ follow-up, and potentially seeking other strategic procedures that may have been effective toward retention in existing research is needed. Published manuscripts often do not reflect the varied strategies employed through the duration of study and, moreover, do not test retention strategies within their study. In fact, as concluded in a recent study via survey and in-depth semistructured interviews, longitudinal studies with high retention rates commonly used personalized approaches and frequently tailored and revised retention strategies specific to participants in their study cohorts [38]. This tailoring is enabled by an understanding of social, cultural, and environmental contexts particular to the population studied.

The research approach chosen by this study has as particular strengths its focus and design. The study focuses on motives for participation and for participants’ reluctance to continue in cohort studies in diverse sociogeographic and cultural settings, while addressing the interplay of the points of view and lived experience of individuals in both researcher and researched stances. Considering both standpoints will allow us to better understand the costs and benefits of different implemented approaches and ultimately contribute to develop novel and more “in-context” strategies to improve retention. The study design resorts to a flexible shared framework for triangulation of various qualitative methods to collect more in-context and nuanced data from diverse sites and involved parties, including a collaborative visual methodology. The potential of collaborative methods and nontextual tools in social science research (and namely in public health and related disciplines) is now also widely recognized and well documented in its ability to evoke more nuanced understanding of the ways in which people experience and perceive their worlds [27,39-42].

As demonstrated by several follow-up studies, preterm birth is associated with neuromotor and cognitive impairments, psychiatric morbidity, and, among others, increased anxiety, social rejection, and reduced self-esteem. When working in settings with communication barriers or with subjects who are more introverted or cannot verbally express themselves because of physical or language difficulties, collaborative visual methods are invaluable additions to researchers’ methodological tools. Another well-documented advantage is their transformative impact on participants. For both researchers and research subjects, they are a powerful and significant means of communicating and discussing ideas while opening up ways of seeing and knowing. Combined with others, collaborative methods can be used to consider the vantage points of different social actors and display complexity and heterogeneity that are critical for understanding social issues. They do not necessarily guarantee a better instrument, yet they do have the potential to provide a larger pool of concepts to be considered in subsequent research development or in interpreting already existing data while building a more participatory relationship.

The study’s main challenge is the use of a situated approach with multiple methods for data collection. It entails increased effort and time while increasing the complexity of analysis. Although not common in health-related research, as mentioned in previous sections, an analysis of multimodal data collected from different sources is established in social sciences as well as the analytical strategy chosen [43]. Data collection and analysis will be thus performed by a multidisciplinary team led by social science researchers experienced in both.

### Conclusions

The culturally sensitive, inclusive, and collaborative approach to public health research on which this study is based expects to inform future research through sharing knowledge about the research process as well as its findings. This study argues for collaboration as a means to empower participants to represent themselves in the research process and in its findings in ways that meet both researchers’ and their own expectations and objectives. We find it the most promising strategy to produce rich multimodal original data, which is inclusive of diverse voices, perceptions, and experiences. We believe that handing over some control to the very people who are providing the data on which findings will be based and acknowledging the value of their participation and involvement will not only enrich the results but also potentiate their engagement and enduring identification with cohort studies.

## Appendix

Multimedia Appendix 1 Interviewing guide of key issues to approach.

## Abbreviations

RECAP: Research on European Children and Adults Born Preterm
VPT/VLBW: very preterm/very-low-birth-weight
